# Development of an addiction recovery patient-reported outcome measure: Response to Addiction Recovery (R2AR)

**DOI:** 10.1186/s13011-023-00560-z

**Published:** 2023-09-01

**Authors:** Elisabeth Okrant, Sharon Reif, Constance M. Horgan

**Affiliations:** Institute for Behavioral Health, Schneider Institutes for Health Policy and Research, Heller School for Social Policy and Management, Brandeis University, MS035, 415 South Street, Waltham, MA 02453 USA

**Keywords:** Substance use disorder, Recovery, Patient-reported outcomes, Measure development, Clinical assessment, Quality measurement

## Abstract

**Background:**

Recovery, a primary goal of addiction treatment, goes beyond abstinence. Incorporating broad domains with key elements that vary across individuals, recovery is a difficult concept to measure. Most addiction-related quality measurement has emphasized process measures, which limits evaluation of treatment quality and long-term outcomes, whereas patient-reported outcomes are richer and nuanced. To address these gaps, this study developed and tested a patient-reported outcome measure for addiction recovery, named Response to Addiction Recovery (R2AR).

**Methods:**

A multi-stage mixed methods approach followed the Patient-Reported Outcomes Measurement Information System (PROMIS) measure development standard. People with lived experience (PWLE) of addiction, treatment providers, and other experts contributed to item distillation and iterative measure refinement. From an item bank of 356 unique items, 57 items were tested via survey and interviews, followed by focus groups and cognitive interviews.

**Results:**

Face validity was demonstrated throughout. PWLE rated item importance higher and with greater variance than providers, yet both agreed that “There are more important things to me in my life than using substances” was the most important item. The final R2AR instrument has 19 items across 8 recovery domains, spanning early, active, and long-term recovery phases. Respondents assess agreement for each item as (1) a strength, and (2) importance to ongoing recovery.

**Conclusion:**

R2AR allows PWLE to define what is important to their recovery. It is designed to support treatment planning as part of clinical workflows and to track recovery progress. Inclusion of PWLE and providers in the development process enhances its face validity. Including PWLE in the development of R2AR and using the tool to guide recovery planning emphasizes the importance of patient-centeredness in designing clinical tools and involving patients in their own care.

**Supplementary Information:**

The online version contains supplementary material available at 10.1186/s13011-023-00560-z.

## Introduction

Addiction, encompassing alcohol and/or drugs, is a chronic disease whereby recovery is often characterized by cycles of remission, recurrence, and treatment. Sustained recovery may take years [1], with significant social and economic impact on individuals, families, communities, and health systems. While remission of substance use is generally considered the start of recovery, the transition to sustained recovery is not well understood. The conflation of abstinence with recovery in clinical and cultural discourse emphasizes remission as an endpoint when, in fact, recovery also requires attention to social determinants of health. With few exceptions [2–4], little is known about the combination of factors that contribute to recovery and, thus, how to measure it.

Pathways to recovery are highly personalized, which makes universal elements difficult to observe. Research to develop such knowledge collectively with people with lived experience (PWLE) of addiction as the primary data source could inform (1) what is critical to ongoing recovery and what threatens it; and (2) whether common elements define recovery. These data could foster deeper insights into sustained recovery, and treatment could focus on resources supportive of these factors. To date, most addiction-related quality measurement has relied upon near-term process measures that are not indicative of long-term recovery or quality of services delivered to support PWLE in their recovery journeys. Further, most have not been developed a priori and conceptually with the goal of clinical integration and intention to capture changes over time. To address these gaps, this study developed and tested a patient-reported outcome measure for addiction recovery with input from both PWLE and providers, named Response to Addiction Recovery (R2AR).

### Defining recovery

Recovery definitions must incorporate a nuanced and multidimensional conceptualization of individual recovery pathways [5]. Definitional efforts have spanned federal agencies, consensus panels, and others [4, 6–8]. One example uses a harm reduction approach to advance recovery science and research: “an individualized, intentional, dynamic, and relational process involving sustained efforts to improve wellness” [9]. The National Institute on Alcohol Abuse and Alcoholism (NIAAA) emphasizes remission from heavy drinking, as well as no longer meeting criteria for an alcohol use disorder, while also acknowledging that “social support and spirituality…physical and mental health, quality of life, and other dimensions of well-being” are critical to ongoing recovery [8].

Existing definitions agree about two main points. First, addressing unhealthy alcohol/drug use and physical dependence is only one of many changes that people in recovery report as important [10]. Therefore, recovery goes beyond remission to a broader view of functional, psychosocial, and environmental well-being [5]. Other key dimensions include coping and functional skills, social connectiveness, physical health, and personal fulfillment [11, 12]. Second, recovery involves a process. The life-course that fostered addiction requires ongoing and transformative change [13–15].

### Recovery: challenges for measurement

The recovery concept is ambiguously deployed in research and clinical practice, mainly because recovery journeys are unique. People must overcome different barriers and benefit from facilitators specific to their individual experience. Their navigation of recovery reflects their history, socioeconomic situations, and perceptions about what constitutes a fulfilling life. A recovery measure must be flexible to capture these individual and changing pathways. To be sensitive to significant and clinically meaningful changes, it must not be constrained by “floor” or “ceiling” effects. With addiction often cyclical and non-linear, a recovery measure will fail to capture progress and outcomes unless this aspect of disease burden is incorporated into its design.

### Individualized measurement via patient-reported outcomes

Patient-centeredness as a quality measurement concept incorporates patients’ voices in defining outcomes [16]. Often contrasted with “objective” tests and clinical assessments (e.g., urine drug screens), patient-reported outcome measures (PROMs) are tools that capture patient perspectives of their health and wellbeing [17]. PROMs allow clinicians to monitor patients’ subjective health status over time and, importantly, function as a warning system if outcomes decline, unlike process measures that focus on early-stage treatment. Yet PROMs must be appropriately developed to incorporate outcomes prioritized by patients, and enable sharing of the information in ways that are relevant to patients [18]. In short, PROMs are patient-centered when developed to measure outcomes patients have defined, rather than reflecting the clinicians’ or researchers’ definition [18, 19]. Additionally, PROMs are patient-centered inasmuch as they invoke shared decision making, so that they empower patients to direct care. This means that PROMs must be acknowledged as part of the treatment process, and the data used to evoke communication between provider and patient.

PROMs can accommodate variation in the recovery definition, its chronic and cyclical nature, and variable and subjective pathways. They allow people to define recovery themselves, reflect subjective changes, and emphasize patients as central in their addiction care, similar to other conditions such as cancer and disability [20, 21], where outcome definitions differ by person and can change over time. Further, given the association between self-efficacy and addiction outcomes [22, 23], PROMs are adaptive to asking PWLE about self-efficacy in the recovery process, to measure and predict outcomes. Shared decision-making may also improve patient-provider relationships and therapeutic alliance, and in turn support treatment plan progress [22]. Despite importance for patient-centered care, a PROM that reliably measures the complexity of recovery, as well as any changes over the course of recovery, does not yet exist for addiction.

### Existing recovery measures

Despite these challenges, eight validated recovery measures exist [24–31], two of which are shortened versions. Table 1 describes these existing recovery measures; Table 2 compares their recovery domains. All capture the fundamental notion that recovery is multidimensional and primarily reflects changes that occur outside of treatment [3, 14, 32, 33]. Yet, gaps remain [34], including the generalizability of the populations tested [35]. Further, the domains and items differ across the existing measures, and none are comprehensive. Okrant [34] offers a comprehensive review of the existing measures developed prior to 2019, summarized and updated with new measures here.

**Table 1:** Elements of existing measures of substance use recovery

Measure	Items (#)	Type of responses	Involved PWLE or Stakeholders	Tested clinically	Domains
**Assessment of Recovery Capital (ARC) ** [24]	50	yes/no	PWLE, Providers	No	(*N* = 10) substance use & sobriety, global psychological health, global physical health, citizenship & community involvement, social support, meaningful activities, housing & safety, risk-taking, coping & life functioning, recovery experience
**Hope and Coping in Recovery Measure (HCRM) **[25]	10	Likert scale	Researchers, experts	Yes	(*N* = 2) hope, coping
**Substance Use Recovery Evaluator (SURE) **[26]	21	Likert scale	PWLE	No	(*N* = 5) substance use, material resources, outlook on life, self-care, relationships
**Recovery Progression Measure (RPM) **[27]	36	yes/no (30 items); Likert scale (6 domains)	SUD Professionals	No	(*N* = 7) lifestyle, thoughts, emotions, behaviors, life situations, physical health, functioning
**Rapid Recovery Progression Measure (Rapid RPM) **[28]	10	Likert scale	SUD Professionals	No	same as RPM
**Brief Assessment of Recovery Capital (BARC-10) **[36]	10	Likert scale	Not beyond original ARC	No	same as ARC
**Brief Addiction Monitor (BAM) **[30]	17	Continuous variable	Researchers, clinician panel	Yes	(*N* = 3) substance use, risk and protective factors
**Multidimensional Inventory of Recovery Capital (MIRC) **[31]	28	4 categorical options: disagree, disagree, agree, and strongly agree	Researchers, PWLE, service providers	No	(*N* = 4) social, human, physical, and cultural capital

*Abbreviations*: *PWLE* people with lived experience of substance use disorders; *SUD* substance use disorder

**Table 2:** Comparison of domains in existing recovery measures

		**Recovery Measure**					
	**Recovery Domains in Existing Measures**	**ARC** [24] **BARC** [28]	**Hope & Coping** [25]	**SURE** [26]	**RPM** [27] **Rapid RPM** [36]	**BAM** [30]	**MIRC** [31]
**Meaning & Purpose**	Meaningful activities	X				X	X
	Outlook on life		X	X			X
**Social Support**	Social support	X				X	X
	Relationships			X		X	X
**Psychological Well-Being**	Global health (psychological)	X			X	X	X
	Self-care			X		X	
**Community Connectedness**	Citizenship, community involvement	X				X	
**Coping & Life Functioning**	Coping & life functioning	X	X		X	X	X
	Emotions				X		
**Health**	Global health (physical)	X			X	X	X
**Recovery Experiences & Risky Behaviors**	Substance use & sobriety	X		X		X	X
	Risk taking	X			X	X	
	Lifestyle				X		
	Recovery experience	X				X	X
**Environment, Housing, & Safety**	Housing and safety	X					X
	Material resources			X		X	X

The Assessment of Recovery Capital (ARC) [24] was the first recovery measure developed. Including 50 items across 10 domains based on biopsychosocial principles, and developed iteratively with patients and providers, it is inclusive of heterogeneous recovery pathways. However, dichotomous yes/no responses lead to a “flattening” of experience gradients and contextual meaning across people who share a particular stratum. Furthermore, the ARC was not designed to link outcomes to interventions, thus has limited clinical use, although a shortened version was created to address this concern. The Hope and Coping in Recovery Measure [25] lacks other important recovery indicators, and was developed using only college-age students.

The Substance Use Recovery Evaluator (SURE) measure used a consumer-driven theoretical framework throughout development, with PWLE contributing to its initial formulation and refinement [26]. Its 21 items fall within five domains, with recovery scores generated by Likert-scale responses, with administration intended to enhance the patient-provider therapeutic relationship by eliciting patient narratives. The Recovery Progression Measure (RPM) was developed to incorporate the link between recovery capital and psychometrically valid outcomes, a limitation of most other tools [27]. Based on Cognitive Behavioral Therapy (CBT), the RPM includes 36 items in 6 domains with demonstrated internal validity. Dichotomous responses are used within domains, along with an 11-point Likert scale to assess each domain overall. However, PWLE were not included in measure construction, thus it lacks patient-centeredness as a core value, and cannot assert face validity.

Two shortened (10-item) recovery measures have been created to reduce burden and be more amenable to use within treatment settings. Vilsaint et al. created the Brief Assessment of Recovery Capital (BARC-10), using item response theory to reduce the items in the ARC [28]. Although the BARC-10 reduces burden, it also offers less information for treatment planning [29]. The Rapid RPM was created to reduce response time and burden, although without PWLE involvement at this stage [36]. It may have the same concerns about sufficient information for treatment planning as noted for the BARC-10. The Brief Addiction Monitor (BAM) instrument developed by the Veterans’ Association is a 17-item instrument that measures subdomains: risk factors for substance use, protective factors for sobriety, and drug and alcohol use [30]. The BAM does not provide a psychometrically valid score [35, 37], but can be used to look at changes across domains.

Finally, the most recent recovery measure, Multidimensional Inventory of Recovery Capital (MIRC) by Bowen et al. [31] has aggregated the conceptual models across many pre-existing measures, and added negative recovery capital, or barriers to recovery, to operationalize their construct. The measure was developed for alcohol use disorder only and might not generalize to other substance use disorders. We note that the International Consortium of Health Outcome Measurement (ICHOM) has recently completed work on measures and related methodological processes for assessing the outcomes of substance use treatment [38], yet this approach is not a unique instrument, thus is not described here.

The measures vary by items and measurement approach. Several ask yes/no questions, which can lead to a “flattening” of experience gradients and contextual meaning across people. PWLE were not included in the development of four measures, which may reduce face validity and patient-centeredness as a core value. Several others did involve PWLE, with the SURE doing so in a most comprehensive fashion [18]. Most were not tested in clinical settings, nor were they psychometrically validated across subgroups and substance use diagnoses. The shortened measures aim to reduce burden and increase feasibility within treatment settings, yet may be too concise for treatment planning. The R2AR instrument described here was designed to more comprehensively address these challenges.

### Study objective

**Fig. 1:**
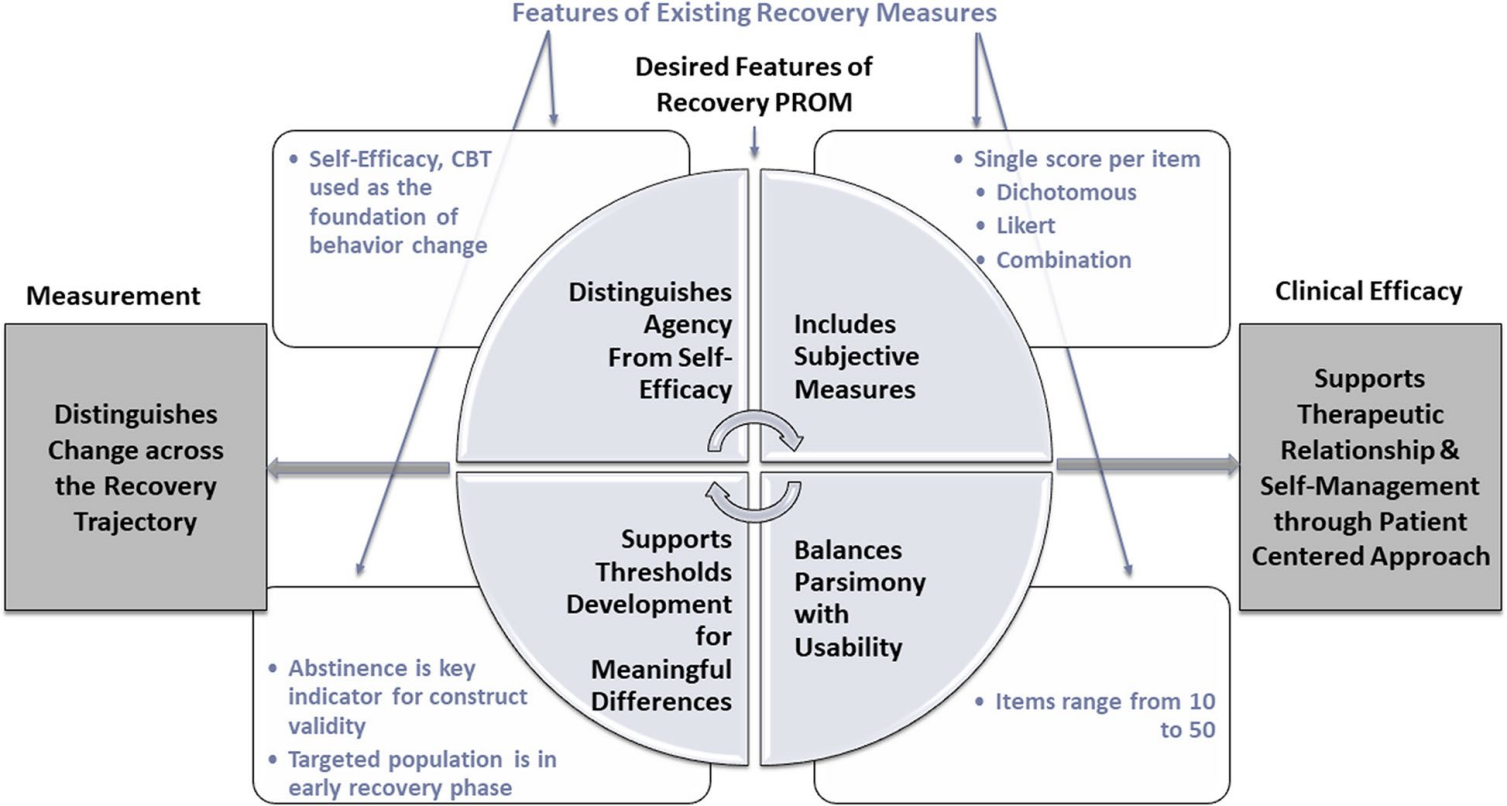
Desired features of recovery PROMs and features of existing recovery measures

This study aimed to develop a PROM for addiction recovery that captured the complexity of recovery; was agnostic to specific types of substances, level of use, or place of service; and incorporated PWLE in development and testing. The R2AR instrument accomplishes this. It aims to be feasible and useful in clinical settings, and relevant beyond specialty addiction treatment settings, including mental health settings and, importantly, primary care settings where standardized assessments are essential to support non-specialists in identifying and treating chronic conditions such as addiction. Currently, primary care might not be offering substance use treatment to individuals regularly, but many stakeholders have advocated for practice transformation wherein specialty care or functions of specialty care are integrated within primary care to offer whole person care [39]. The R2AR instrument would support primary care in monitoring progress of those patients they treat who have substance use disorders, even acting as an indicator that referring out might be warranted in the event that patients are not exhibiting progress. The R2AR should address limitations of existing measures and set the stage for patient-centered and measurement-based care in addiction treatment settings (see Fig. 1). This paper shares the methods used to develop and refine the R2AR.

## Methods

**Fig. 2:**
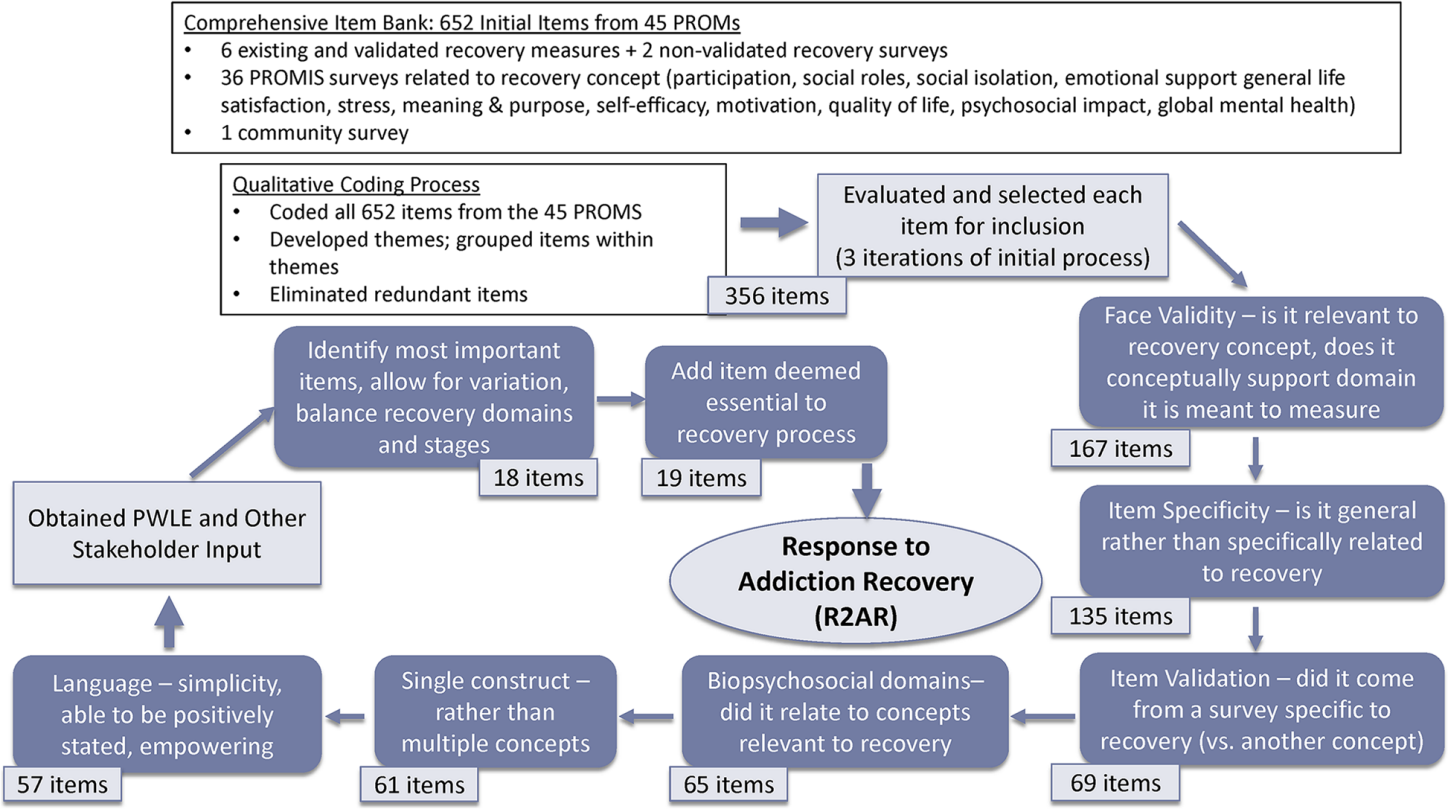
Item Reduction Process and Criteria

The study relied primarily upon qualitative methods, which are particularly important in establishing patient-centered PROMs with face validity for the addiction treatment seeking population [40]. The process included: (1) developing an item bank following the PROMIS method [41]; (2) distilling, testing, and refining items through an iterative mixed methods approach, including PWLE and behavioral health (BH) providers; and (3) testing with patients and providers at a non-profit federally qualified health center (FQHC). The first two phases are more fully described by Okrant [34]. This process is summarized in Fig. 2 (item reduction) and Table 3 (stakeholder input), with additional detail below. The research was approved by the University’s Institutional Review Board. All participants provided informed consent, including for audio recording.

**Table 3:** Response to Addiction Recovery (R2AR) development—stakeholder input process

Task	Description	Sample and Honoraria	N
**Initial Item Review**			
1. Stakeholder survey (online)	Identify items important to recovery, assign recovery domain and stage of recovery. Each participant reviewed a randomly selected 28 items of the 57 items, to reduce participant burden	PWLE, BH/medical/social service providers, researchers, recovery coaches/peers, family/friends of people in recovery. Lottery for one of four $50 gift cards	*N* = 330
2. PWLE interviews (phone or video)	Determine if items captured recovery experiences, missing concepts, irrelevant items, user experience	PWLE survey respondents who agreed to be contacted (N = 30); snowball method (N = 2). $50 gift card	*N* = 8
**Cognitive Testing**			
3. PWLE interviews (phone)	Complete revised R2AR, provide feedback on meaning, usability, usefulness, user experience	PWLE who completed initial interview and willing to be interviewed. $50 gift card	*N* = 8
4. BH provider (in-person or email)	Assess whether items captured range of recovery progress and stage, item clarity and meaning, Likert scale, anchor questions	Convenience sample of BH providers. $50 gift card (offered but none accepted)	*N* = 5
5. Expert review (in-person)	Assess anchor items, Likert scale, items and domains	Authors, researchers, and clinical colleagues at BH-focused FQHC	*N* = 6
6. Clinical focus group (in-person)	Determine if items were important to clinical practice, appropriate for OUD/MOUD population, viable for clinical setting	Nurse care managers who support people receiving MOUD at BH-focused FQHC. $25 gift card	*N* = 6
7. Client focus group (in-person)	Meaning of items, comprehension, relevancy to own recovery experience. Viability of items, anchors and Likert scales for measuring recovery	Individuals treated for addiction at BH-focused FQHC. $25 gift card + lunch	*N* = 25
8. Client interviews (in-person)	Review items/scale/anchors, discuss meaning, thought process for response	Individuals treated for addiction at BH-focused FQHC, recruited for focus groups but did not participate due to capacity. $25 gift card	*N* = 4

*Abbreviations*: *BH* behavioral health, *FQHC* federally-qualitied health center, *MOUD* medications for opioid use disorder, *OUD* opioid use disorder, *PWLE* people with lived experience

### Initial R2AR Development

R2AR construction used the Patient-Reported Outcomes Measurement Information System (PROMIS) for measure research and development recognized by the Centers for Medicare & Medicaid Services (https://mmshub.cms.gov/) [42, 43]. A comprehensive item bank was developed from existing measures [41], including 45 measures and their 652 individual items. All items were imported into Atlas.ti. v.8.4.4, then coded to identify unique concepts using inductive grounded theory [44].

The infrastructure to categorize items was developed from eight recovery domains identified from the literature and existing recovery measures, with review by five expert addiction treatment and recovery researchers and clinicians to confirm relevance to the recovery construct. These domains include meaning and purpose, psychological well-being, environment/housing/safety, social support, coping and life functioning, recovery experience/risk taking, health, citizenship and community involvement.

### Item bank distillation process

Distilling the item bank was iterative. (1) Initial coding and item reduction by the first author (EO). (2) A survey with PWLE, who self-identified as being in recovery from any type of substance use problem, and other stakeholders for further item reduction and sorting by recovery domains and stages. (3) In-depth interviews with a subset of survey respondents. (4) Cognitive testing interviews with PWLE and behavioral health (BH) providers. Figure 2 illustrates the item reduction process. The expert input into selecting existing measures and initial distillation, as recommended in the PROMIS method, was balanced with PWLE input via qualitative methods immediately following this initial stage, including the opportunity to recommend any concepts that were omitted.

#### Initial item bank coding and reduction

After removing duplicates, 356 items were assessed for overlapping meaning. Core categories were refined by removing redundancies and items that did not fit, then combined into larger theoretical themes. Items with little face validity or likely relevance to recovery outcomes were removed.

#### Survey of PWLE and stakeholders

The online survey solicited feedback about the 57 remaining items to ensure that PWLE were part of developing and validating domains. Respondents were asked which items were most important to their own recoveries (or of people known to them) on a 1 to 10 scale, and to assign each item to a recovery domain and stage. The recovery stage question aimed to capture changes in recovery over time, to inform clinical recovery pathways and understand transitions from early to active to long-term recovery. The placements of each item into one recovery domain provided insight into how they perceived the underlying constructs, approximating a data-driven factor analysis.

Responses (*N* = 330) were primarily PWLE (*N* = 188) and BH providers (*N* = 94) (see Supplemental Table A1). These groups were examined separately and in comparison, using a correlation matrix to identify items with similar constructs, and Cronbach’s alpha to test the internal consistency of items within domains, without defining a dimensional structure during this initial testing. The remaining survey participants (*N* = 48) were family/friends or other stakeholders, who were dropped from this analysis.

#### Interviews with PWLE

PWLE survey respondents also were invited to share their recovery experiences through qualitative interviews. Structured open-ended questions aimed to understand if the initial items captured their recovery experiences, and identify missing concepts or less relevant items. Interviewees were asked about the experience of responding to the items (e.g., triggering language or positive/negative feelings).

Interviews were coded via grounded theory. Codes were examined across interviews to identify similar underlying meaning, and renamed as appropriate. Ultimately, 31 interview-based codes emerged (Supplemental Table A2). No new codes were identified; rather interviews provided confirmation and guidance for further reduction of items.

#### Iterative development of the R2AR

The 57 items tested in the survey and 31 interview codes were then examined together. Balance across domains and response variation were considered. That is, people differed on which items were or were not most important to their recoveries or items that were less important. These distinctions were examined to ensure sensitivity to varied recovery experiences and changes across stages. Therefore, some items not uniformly thought to be critical to recovery were retained. Some items were modified to enhance clarity. The resulting preliminary R2AR instrument included 18 items.

#### Cognitive testing interviews

The original PWLE interview participants agreed to complete the preliminary R2AR and were re-interviewed regarding how well the instrument captured individual recovery experiences; and whether it promoted any positive/negative feelings, experiences, etc.

Five BH providers provided insight into how the preliminary R2AR might be further refined. They assessed whether the items captured recovery progress and were representative across recovery stages; item clarity and meaning; the Likert scale; and the value of asking people in recovery about the relative importance of each item to their recoveries. The resulting revised R2AR was tested in a clinical setting.

### R2AR refinement within a clinical setting

Further revisions stemmed from preparations for a pilot clinical trial to validate the R2AR among people receiving medications to treat opioid use disorder (MOUD) (NCT05388045). The authors and clinical partners at an addiction-focused Federally Qualified Health Center (FQHC) reviewed the instrument, refining it to more accurately represent recovery domains and ensuring the anchor questions and Likert scale optimized precision and variability within and across people during different recovery stages.

At the same clinical setting, focus groups were held with nurse care managers (NCMs) who support clients on MOUD and, separately, with a group of addiction treatment clients, followed by additional cognitive interviews of addiction treatment clients. The addiction treatment clients were recruited from the addiction treatment programs at this clinical partner.

#### Nurse Care Manager (NCM) focus group

The NCM group discussed whether the items addressed recovery areas they deemed important to clinical practice and its viability for clinical use. They were asked to suggest alternatives to items that they questioned, to move closer to the intended meaning.

#### Client focus groups

Individuals treated for addiction at the FQHC were recruited for two focus groups. Participants discussed interpretation of each item, dissecting what it was trying to capture about recovery. They explained how they comprehended the item, and relevance to their own experiences. As needed, the group collectively edited items until consensus was reached. The group setting offered a unique opportunity to shape the items in alignment with their varied lived experiences and meaning. Participants also considered whether the items, anchor questions, and Likert scales were viable for measuring the recovery construct. The R2AR incorporated feedback from all three focus groups, then was reviewed by the research team.

#### Cognitive interviews

Again, cognitive interviews with addiction treatment clients aimed to assess concordance with intended item meaning and the cognitive approach that resulted in a particular response. The participant reviewed each item, then explained their perceived meaning and their thought process when deriving a response; discussion included the Likert scale and anchor questions.

### Response to Addiction Recovery (R2AR) instrument

The research team revised the R2AR a final time prior to fielding in the pilot clinical trial. In summary, constructing the R2AR required balancing four components: (1) Prioritizing items that PWLE and stakeholder groups expressed were most important recovery, while allowing for variation. (2) Distributing items across early, active, and long-term recovery. (3) Representing all eight recovery domains. (4) Creating face validity via iterative process with PWLE and stakeholders, and construct validity using qualitative codes to ensure no gaps existed among the items conceptualizing the recovery construct. The final R2AR instrument is described below.

## Results

### Survey of PWLE and Stakeholders

#### Survey participants

The survey was completed by *N* = 330 people overall, with the majority (*N* = 188) PWLE and (*N* = 94) BH providers. Item response rates ranged from 84.0% to 90.5%.

PWLE survey respondents had a broad range of substance use, recovery, and treatment experiences. The vast majority were in recovery for 2 + years, with 80% in long-term recovery. Most reported 10 + years of substance use problems before their current recovery, most with multiple substances; alcohol was most frequently the primary substance, followed by heroin and other opioids. About 60% reported mental health issues. To achieve their most recent recovery period, most (80%) received formal treatment. Almost half the states were represented, with most from Massachusetts, followed by California, Michigan, and Pennsylvania. (See Supplemental Table A3 for sociodemographics and Table A4 for recovery, substance use and mental health characteristics.)

Among BH providers, about 37% had a professional degree (e.g., MSW), and nearly 9% had a certificate or training (e.g., Certified Alcohol and Drug Counselor). Most were from California or Massachusetts. (See Supplemental Table A5 for sociodemographics.)

#### Survey results - ranking of items important to recovery

Overall, PWLE rated item importance higher and with greater variance than BH providers (Table 4). This suggests that PWLE responded for their individual pathways, whereas BH providers were more likely to respond through the lens of treating multiple people, i.e., aggregating across their experiences.

**Table 4:** Importance rankings of preliminary R2AR items—people with lived experience and behavioral health providers

	**Mean Importance Rating** **(1 to 10 scale)**		
**Item (ranked by PWLE mean rating)**	**PWLE**	**BH Provider**	**Difference**
1. There are more important things to me in life than using substances	9.44	9.50	-0.06
2. I can manage conflict with a partner, relative or friend without turning to alcohol or drugs	9.38	8.83	0.55
3. I take full responsibility for my actions	9.29	8.91	0.38
**4. It is important that I make a contribution to society**	**9.11**	**7.52**	**1.59**
5. I will get better	9.10	8.41	0.69
6. I am making good progress on my recovery journey	9.00	8.13	0.87
7. I am aware of the love and support available from other people	8.94	8.22	0.72
8. I have people around me who know how to get through life without using alcohol or drugs	8.91	8.58	0.33
**9. I am getting along with family or friends better than I did before**	**8.89**	**7.50**	**1.39**
10. I have a network of people I can rely on to support my recovery	8.81	8.80	0.01
11. I can manage to solve difficult problems if I try hard enough	8.78	8.17	0.61
12. I am free of threat or harm when I am at home	8.77	8.87	-0.10
13. I am actively engaged in efforts to improve myself (training, education and /or self-awareness)	8.72	7.98	0.74
14. I have a good sense of what makes my life meaningful	8.72	8.69	0.03
**15. I make sure I do nothing that hurts or damages other people**	**8.70**	**7.79**	**0.91**
16. I am taking care of my mental health more now than I did before I started focusing on my recovery	8.66	8.09	0.57
17. I appreciate that I am part of the universe and something bigger than myself	8.66	7.85	0.81
18. I feel close to people I care about	8.61	8.09	0.52
19. My relationships are meaningful	8.57	8.37	0.20
20. I am able to enjoy life	8.56	7.98	0.58
21. I feel I am in control of my substance use	8.49	7.87	0.62
22. In general I am happy with my life	8.45	7.61	0.84
23. I have a realistic appraisal of my abilities and my limitations	8.32	7.43	0.89
24. I have access to opportunities for career development	8.31	7.82	0.49
25. I feel determined	8.29	8.05	0.24
26. I am comfortable with who I am	8.28	8.09	0.19
27. I am satisfied with my ability to do the work that is really important to me (include work at home)	8.28	7.87	0.41
28. I can adjust to things I cannot change	8.24	8.12	0.12

Bolded rows indicate the 3 items with the greatest difference in means, by at least .9 point, comparing People with Lived Experience (PWLE) to Behavioral Health (BH) providers

The 57 items were moderately to highly correlated (range 0.41 to 0.88 for PWLE, 0.60 to 0.99 for BH providers) indicating similarity of concepts and highlighting items for elimination. Cronbach’s alpha (0.97) confirmed internal consistency.

Both PWLE and BH providers agreed that *“There are more important things to me in my life than using substances”* was the most important item for recovery, with a mean “importance to recovery” ranking of 9.44 (standard deviation 1.54) out of 10 points by PWLE and 9.50 (standard deviation 0.82) by BH providers. The item emphasizes that self-efficacy, not abstinence, was central to recovery pathways.

#### Survey results - recovery stages and recovery domains

PWLE and BH providers responded similarly for recovery domains and stages for most items. The items were fairly well-distributed across domains, although most were in the “Meaning and Purpose” and “Psychological Well-Being” domains. Items were also well-distributed across all recovery stages. PWLE selected long-term recovery for more items than BH providers, who selected active recovery for more items; early recovery items were comparable across the two groups. For some items, multiple stages of recovery applied. Table 5 summarizes items by domain and stage.

**Table 5:** Final items included in the Response to Addiction Recovery (R2AR), by Recovery Domain and Stage

Item^a^	Recovery Domain	Recovery Stage
1. There are more important things to me in life than using alcohol or drugs	Meaning & Purpose	Early
2. I feel close to people – like friends, someone I met through recovery or recovery meetings	Community Connection	Early, Active, Long-term
3. I can handle stress, conflict, and unexpected things without alcohol or drugs	Coping	Early
4. I do things, even when I do not want to, because I know that I should	Meaning & Purpose	Active
5. I have activities and hobbies that keep me busy	Meaning & Purpose	Early, Long-term
6. I feel safe in my living environment (i.e., home, shelter, street)	Environment, Home, Safety	Early
7. I am taking care of my physical health	Health	Long-term
8. I feel like I am in control of what happens in my life	Recovery Experiences	Early, Active
9. I am trying to improve myself (by learning a trade or skill, going back to school, or any kind of self-help)	Meaning & Purpose	Active
10. I am taking care of my mental health	Health	Early, Active, Long-term
11. Most times, I do not worry about what other people think of me (because of my past drug use)	Psychological Well-being	Early, Long-term
12. Through the recovery process, I realized that I have good qualities (examples: I am a good person/parent/son/daughter/ husband/wife, friend, hard worker, help others)	Recovery Experiences	Early, Active
13. I am dealing with my legal problems (like custody, warrants, paying fines or child support)	Coping	Early
14. I try not to hurt other people with my actions	Recovery Experiences	Early, Long-term
15. There are people who care about me who I trust (like my therapist or clinician, a sponsor, friends, or family), who I can turn to for help during difficult times in my recovery	Social Support	Early, Active, Long-term
16. I have what I need to work on future goals (such as money, a way to get around, housing, food)	Environment, Home, Safety	Active
17. I feel less shame than I did before about my past	Recovery Experiences	Long-term
18. I feel like I am part of a larger community (such as people in my neighborhood, at work, or church)	Community Connection	Early
19. I am hopeful	Recovery Experience	Early, Active, Long-term

R2AR instrument is available from the authors

^a^Each item is scored on a 5-point Likert scale, within the structure of two parent questions. (1) In general, how much do you agree with the following statement [i.e., item]? (2) How important is this [item] to you to work on?

### Iterative selection and revision of themes and items for inclusion

The emergent themes identified through the interviews were consistent with the eight recovery domains and survey responses. During the NCM focus group, an additional item, “I am hopeful”, was strongly recommended, and included for final cognitive testing.

To capture the complexity of recovery outcomes over time, it was important for final items to be included across stages and domains. Some high-ranking items were ultimately excluded due to over-emphasis of a recovery stage or domain and to allow for inclusion of other key items. For example, the item “I feel connected to my community” [45] was retained despite lower ranking because it could lead to interventions that support reintegration into social networks. Further, it was significantly endorsed through interviews and focus groups that linked community connectedness to recovery.

### Clarifying wording and meaning

The cognitive interviews validated items’ intended meanings with one exception: *“I am dealing with my legal problems (like custody, warrants, paying fines or child support).”* PWLE answered “strongly agree,” but regarding past problems while the question was intended for current issues. Their pride for remediating downstream addiction issues was considered still relevant, so their responses reflected the item’s importance to their present recovery. The item was retained.

### Final item selection and balance

The final R2AR contains 19 items, balanced across the three stages of recovery and eight recovery domains (Table 5). For each item, the R2AR asks, on a 5-point Likert scale: (1) In general, how much do you agree with the following statements, and (2) how important is this to you to work on? The R2AR instrument is available from the authors.

## Discussion

This study aimed to develop an addiction recovery PROM tool that could be feasibly administered, support providers in measuring clinically meaningful progress in recovery, and strengthen the therapeutic relationship by focusing on what matters to people in recovery. The R2AR instrument developed here systematically captures the essential recovery domains and comprehensively incorporates perspectives of PWLE. It is the first to measure recovery through early, active, and late stages, allowing for sensitivity to changes across time, and uses a 5-point scale for each item, potentially a larger floor and ceiling to capture those changes. This tool aligns with the National Quality Forum guidebook [46] to simultaneously develop PROMs with performance measures to drive high-quality measurement-based clinical care. In other words, capturing items across the spectrum of recovery progress will allow quantification which, in turn, will allow for capture of clinically meaningful change.

As noted, a challenge to measuring recovery is that it is multidimensional, individually experienced, and frequently in flux. To date, measures have not accounted for dynamic movement. The R2AR uniquely uses both subjective and objective measures so that individuals can dynamically define what is important to them at a given time. The R2AR does not intrinsically apply an overarching preconceived idea of recovery, which would falsely suggest that research and clinical practice fully know what defines recovery and for whom. It moves toward a flexible approach that allows individuals’ definitions of recovery to change over time. We requested that PWLE define what “early,” “active,” and “long-term” recovery mean to them to analyze overlapping themes for these commonly used terms without accepted clinical definitions. These phases are important to define to monitor progress in recovery and quantify quality of care. Traditional definitions using time in remission or time without using substances are insufficient to explain how people move along the continuum of care or progress in recovery. While there is a potential of recall bias among the respondents, methods that rely upon people’s interpretation of their own experiences are critical to developing measures that matter to PWLE. Additionally, we were able to confirm the validity of these findings through convergent validity with providers’ view of these phases, which were consistent with PWLE.

Through our qualitative interviews and focus groups, we learned from patients that the value of assessments is in their ability to evoke discussions with providers, and for providers to acknowledge the responses patients provided in these surveys. In other words, simply completing the instrument does not benefit patients (though it might via the Hawthorne effect); the utility is in surfacing the patient voice to the provider. As such, the instrument is not merely meant to be a data point, but rather a means for eliciting narratives from the patient that enable patient-directed care. Given responses to R2AR, the provider would help guide the patient to determine what aspects of recovery are most relevant to work on and implement a care plan to support those goals.

The strength of this instrument development is in the multi-phased, mixed methods approach and PWLE focus, and reliance on the gold standard for iterative data collection and refinement. The R2AR built upon previous knowledge in the addiction treatment and recovery field and applies learnings from other fields that effectively use PROMs. If further validated, it could have enhanced clinical utility over prior measures because of its potential to support integrated care models for patients with any SUD, and balances brevity with a range of information that can assist recovery planning. The R2AR was developed for use in any setting, including primary care, similar to other standardized assessments (e.g., depression screening) which support non-specialists and care coordinators in managing chronic disease. Additionally, we carefully crafted the language of each item and tested items with PWLE to ensure that the language was not triggering, remained positive, and did not reify negative self-stigma. We believe that this characteristic of R2AR is uniquely patient-centered.

Testing for usability and sensitivity continues, along with psychometrics more broadly. We will continue to pilot R2AR among different subgroups to ensure sensitivity across subpopulations, within treatment and care settings that are embedded in communities of people with various sociodemographic backgrounds and who experience different ecological impacts [47], prior to a larger trial. Ongoing work is also examining the feasibility of using the R2AR to assess changes over time. We expect that the tool will be demonstrated as agnostic to specific types of SUD to make it easier to implement across PWLE and within clinical settings and easier to aggregate for a quality of care measure. If we learn that the three stages of recovery that we initially partitioned as early, active, and long-term are not representative across subgroups, future efforts will also address variation in experiences when determining which items are indicative of recovery stages across groups.

The next step is to use the subjective (what is important) and objective (how much do you agree) questions to create a scoring methodology and usable “report card” that prioritizes the items that individuals feel are most important to their respective recoveries. The score will go beyond summation of Likert scale points, to account for individual perspectives and measure progress based on achievement toward their important items. For example, scoring of the “subjective” items about what is important for the person to work on could be compared over time and scores used in clinical conversation about why focus changed (i.e. because goals were met and new items have elevated in importance, or if biopsychosocial changes in their environment have altered the way in which people think about what is best for their recovery plans), as well as how to meet new goals. An overall score, weighted by patient assessment of items important to recovery, could be used as an overall assessment of progress on individualized recovery plans that is meaningful for that that person. As we learn more about which items belong to early, active, and long-term recovery, we will aim to develop minimal clinically important differences, which is another tool for evaluating recovery progress. From a patient panel perspective, providers could learn about their operational and treatment effectiveness. For example, they would be able to observe at a practice level, how effective they are at addressing certain aspects of recovery. They could create action plans to improve quality of care and service based on the items that they are least able to improve.

Ultimately, a primary goal of this PROM is to have a reliable tool that will assess personal recovery progress in a patient-centered manner and measure quality of treatment when aggregated across a patient panel, and reflect clinical utility within existing workflows. Our approach to meet this goal is consistent with the ICHOM methodology [38].

Several limitations should be noted. The initial item bank development and refinement was completed by the first author, an expert in quality measurement and patient reported outcomes, without PWLE input. As a more objective process to identify all relevant metrics, reduce duplicates, and identify unique concepts, results from this phase were less likely to be uniquely driven by PWLE input and our approach reduced their burden. However, the iterative process with PWLE through focus groups and interviews provided opportunities for new items to be added. For example, “I do things, even when I do not want to, because I know I should,” was an item that was added resulting from the qualitative interviews. PWLE respondents in the survey and first set of interviews were mostly in later stages of recovery and involved with a recovery community (e.g., mutual-help). Responses might not generalize to people in earlier recovery or not involved in 12-step programs. However, later interviews were with people active in treatment, broadening our base; these participants were treated for addiction at one FQHC thus responses might be specific to that experience, and might not reflect the sensitivity for all SUD types. All PWLE were volunteers, so might be different from those who chose not to participate. The PWLE population was highly educated, employed, and mostly white, thus results may not reflect the broader population in addiction recovery. Work is underway to capture a Black/African-American population in a lower-income urban area. This initial study emphasized development and face validity of the R2AR; future work will examine psychometrics.

The methods used to develop the R2AR ensure face validity which is critical to capturing the recovery construct; the ongoing pilot study will evaluate whether there is a factor structure that supports the conceptual recovery domains. It also will assess the feasibility of the R2AR in clinical practice, determine if the instrument has value to clinicians and people receiving MOUD, and capture changes over time. It will collect reliability and validity data, including correspondence with self-efficacy, health-related quality of life, therapeutic alliance and substance use itself.

## Conclusion

This study tested a new concept for recovery PROMs that captured the complexity of addiction recovery and allowed for patient feedback on their important goals to work on specific to their recovery journey. This R2AR instrument embedded in its design the notion of self-directed care which brings together measurement-based care, patient-directed care, and therapeutic alliance into a single clinical instrument.

The purpose of developing an addiction recovery PROM is threefold: (1) to aid PWLE in self-directing treatment and establishing agency to track recovery, as well as evaluate recovery progress from an individual perspective; (2) to facilitate the treatment relationship, and actively monitor whether interventions to address recovery elements, identified as important by PWLE, are in fact improving; and (3), to support integration, efficiency, and appropriate referral decision making within primary care settings. Clinical tools that support the therapeutic relationship and capture objective measures of change over time, allowing patients the agency to report on progress, must be developed while considering patient and provider burden, feasibility, and usability. For health systems to transform and serve vulnerable populations like the addiction recovery population, innovative approaches to data collection and measurement are required. For that reason, the PROM developed here, Response to Addiction Recovery (R2AR) offers an approach to reporting outcomes that accounts for differences across people and encourages them to assess their recovery progress according to their own recovery definitions.

### Supplementary Information

**Additional file 1:** **Table A1. **Survey Participants by Type. **Table A2. **Emergent Codes from Interviews. **Table A3. **Self-Reported Sociodemographics of PWLE Survey Respondents. **Table A4. **Self-Reported Recovery, Substance Use and Mental Health of People with Lived Experience (PWLE) Survey Respondents. **Table A5. **Self-Reported Sociodemographics of Behavioral Health Provider Survey Respondents.  

## Data Availability

Data generated from this study are not publicly available, due to restrictions required to protect the privacy of participants. The Response to Addiction Recovery (R2AR) instrument that was developed through this work is available upon request from the authors.

## Abbreviations

BH: Behavioral health
FQHC: Federally Qualified Health Center
MOUD: Medications to treat opioid use disorder
NCM: Nurse care managers
PROM: Patient-reported outcome measure
PWLE: People with lived experience of addiction
R2AR: Response to Addiction Recovery instrument
SUD: Substance use disorder
