# Supplementary material for: Development of an addiction recovery patient-reported outcome measure: Response to Addiction Recovery (R2AR)
Source: Subst Abuse Treat Prev Policy. 2023 Sep 1;18:52. doi: 10.1186/s13011-023-00560-z (PMC10474628; doi:10.1186/s13011-023-00560-z)
Supplement: Supplementary file 1 — Additional file 1: Table A1. Survey Participants by Type. Table A2. Emergent Codes from Interviews. Table A3. Self-Reported Sociodemographics of PWLE Survey Respondents. Table A4. Self-Reported Recovery, Substance Use and Mental Health of People with Lived Experience (PWLE) Survey Respondents. Table A5. Self-Reported Sociodemographics of Behavioral Health Provider Survey Respondents. [file 13011_2023_560_MOESM1_ESM.docx]

Development of An Addiction Recovery Patient-Reported Outcome Measure: Response to Addiction Recovery (R2AR) – Supplemental Materials

**Table A1: Survey Participants by Type**

| Type of Participant | Number | Percent |
| --- | --- | --- |
| Person in recovery (PWLE) | 188 | 57.0% |
| Behavioral health (BH) provider | 86 | 26.1% |
| Friend/family of someone in recovery | 26 | 7.9% |
| Social service provider | 10 | 3.0% |
| In treatment, not in recovery | 6 | 1.8% |
| Medical provider | 4 | 1.2% |
| Researcher | 3 | 0.9% |
| Not in treatment, not in recovery | 1 | 0.3% |
| Other | 6 | 1.8% |
| **Total** | **330** | **100%** |

**Table A2: Emergent Codes from Interviews**

| Recovery Domains | Qualitative Codes |
| --- | --- |
| Meaning and Purpose | Forming a relationship with a higher power has given me strength |
|  | I get to be who I am |
|  | I am thinking about what I want my life to look like and I am taking steps toward those goals |
| Social Support | Jail was/was not a motivating factor to stop substance use |
|  | I have stronger relationships now than I ever did before |
|  | I have people I can lean on |
|  | I am checking in with someone regularly about how I am in doing in my recovery |
| Psychological Well-Being | I deserve to be happy |
|  | I am no longer motivated by fear |
|  | I do not use negative words to describe myself |
|  | People see me in the positive way that I see myself |
| Community Connectedness | I feel connected to people in my community |
| Coping & Life Functioning | I look for solutions to problems that are going to help the situations |
|  | I feel in control of my life |
|  | I can handle it when things do not go the way I thought they would |
|  | I have reduced my self-centered behavior |
|  | I am no longer letting myself and other people down |
|  | I manage to show up even when I do not want to |
| Health | I am dealing with the struggles that caused my substance use |
|  | I am getting help with my trauma as part of my recovery |
| Recovery Experiences & Risky Behaviors | Shame is an ongoing struggle |
|  | I accept my limitations |
|  | I feel a new sense of freedom because of my recovery |
|  | I am making good choices |
|  | I am satisfied with myself now despite the flaws of my past |
| Environment, Housing, & Safety | I can support myself [and family] financially without the help of others |
|  | I have a place to live of my own |

**Table A3: Self-Reported Sociodemographics of PWLE Survey Respondents**

|  |  | **N** | **%** |
| --- | --- | --- | --- |
| **Age Group** | 18 – 24 | 2 | 1.87% |
|  | 25 – 34 | 19 | 17.76% |
|  | 35 – 44 | 19 | 17.76% |
|  | 45 – 54 | 26 | 24.30% |
|  | 55 – 64 | 28 | 26.17% |
|  | 65+ | 13 | 12.14% |
|  | *Total* | *107* | *100%* |
| **Race/Ethnicity** | White | 92 | 85.19% |
|  | Black or African American | 11 | 10.19% |
|  | Hispanic | 6 | 5.56% |
|  | American Indian or Alaska Native | 6 | 5.56% |
|  | Native Hawaiian or Pacific Islander | 1 | 0.93% |
|  | Asian | 0 | 0.00% |
|  | Other | 3 | 2.78% |
|  | *Total* | *108* | *100%* |
| **Gender** | Male | 49 | 46.23% |
|  | Female | 57 | 53.77% |
|  | *Total* | *106* | *100%* |
| **Place** | Urban | 50 | 46.73% |
|  | Suburban | 43 | 40.19% |
|  | Rural | 14 | 13.08% |
|  | *Total* | *107* | *100%* |
| **Educational Attainment*** | Less than high school | 0 | 0.00% |
|  | High school graduate | 13 | 12.26% |
|  | Some college | 41 | 38.68% |
|  | 2-year degree | 13 | 12.26% |
|  | 4-year degree | 33 | 31.13% |
|  | Professional degree or Doctorate | 23 | 21.70% |
|  | Vocational school | 2 | 1.89% |
|  | Certificate/training (please specify) | 25 | 23.58% |
| **Employment Status*** | Employed full time | 73 | 68.22% |
|  | Volunteer full time/part time | 21 | 20.39% |
|  | Employed part time | 20 | 18.69% |
|  | Student | 15 | 14.02% |
|  | Unemployed looking for work | 8 | 7.48% |
|  | Unemployed not looking for work | 1 | 0.93% |
|  | Other (e.g., disabled, retired, homemaker) | 20 | 18.70% |
| **Insurance Type*** | Commercial | 61 | 58.65% |
|  | Medicaid | 21 | 20.19% |
|  | Medicare | 20 | 19.23% |
|  | Other | 16 | 15.38% |
|  | None | 2 | 1.92% |
|  | Don't know | 1 | 0.96% |

*** Not mutually exclusive**

**Table A4: Self-Reported Recovery, Substance Use and Mental Health of People with Lived Experience (PWLE) Survey Respondents**

|  |  | **N** | **%** |
| --- | --- | --- | --- |
| **Recovery phase** | Early recovery | 7 | 3.74% |
|  | Active recovery | 32 | 17.11% |
|  | Long-term recovery | 148 | 79.14% |
|  | *Total* | *187* | *100%* |
| **Duration of substance use problems before recovery** | less than 6 months | 2 | 1.77% |
|  | 6 months to less than 1 year | 7 | 6.19% |
|  | 1 year to less than 2 years | 9 | 7.96% |
|  | 2 years to less than 3 years | 12 | 10.62% |
|  | 3 years to less than 5 years | 13 | 11.50% |
|  | 5 years to less than 10 years | 17 | 15.04% |
|  | 10 years or more | 53 | 46.90% |
|  | *Total* | *113* | *100%* |
| **Experienced relapse(s)** | Yes | 59 | 52.21% |
|  | No | 54 | 47.79% |
|  | *Total* | *113* | *100%* |
| **Number of relapse(s) experienced** | 1 | 20 | 33.90% |
|  | 2 to 5 | 27 | 45.76% |
|  | 6 to 9 | 4 | 6.78% |
|  | More than 10 | 7 | 11.86% |
|  | Prefer not to say | 1 | 1.69% |
|  | *Total* | *59* | *100%* |
| **Problems with more than one substance** | Yes | 90 | 79.65% |
|  | No | 23 | 20.35% |
|  | *Total* | *113* | *100%* |
| **Primary substance** | Alcohol | 51 | 45.13% |
|  | Heroin | 22 | 19.47% |
|  | Opioids | 12 | 10.62% |
|  | Cocaine | 10 | 8.85% |
|  | Marijuana | 6 | 5.31% |
|  | Other | 12 | 10.62% |
|  | *Total* | *113* | *100%* |
| **Secondary substance (if applicable)** | Cocaine | 31 | 27.93% |
|  | Alcohol | 22 | 19.82% |
|  | Marijuana | 13 | 11.71% |
|  | Opioids | 8 | 7.21% |
|  | Heroin | 4 | 3.60% |
|  | Fentanyl | 1 | 0.90% |
|  | Other | 11 | 9.91% |
|  | N/A | 21 | 18.92% |
|  | *Total* | *111* | *100%* |
| **Mental health issue** | Yes | 64 | 60.95% |
|  | No | 34 | 32.38% |
|  | Don't know/prefer not to answer | 7 | 6.66% |
|  | *Total* | *105* | *100%* |

**Table A5: Self-Reported Sociodemographics of Behavioral Health Provider Survey Respondents**

|  |  | **N** | **%** |
| --- | --- | --- | --- |
| **Age** | 18 – 24 | 2 | 2.15% |
|  | 25 – 34 | 16 | 17.20% |
|  | 35 – 44 | 17 | 18.28% |
|  | 45 – 54 | 18 | 19.35% |
|  | 55 – 64 | 25 | 26.88% |
|  | 65+ | 15 | 16.13% |
|  | *Total* | *93* | *100%* |
| **Race** | White | 64 | 84.21% |
|  | Black or African American | 3 | 3.95% |
|  | Hispanic | 8 | 10.53% |
|  | American Indian or Alaska Native | 5 | 6.58% |
|  | Asian | 4 | 5.26% |
|  | Native Hawaiian or Pacific Islander | 2 | 2.63% |
|  | Other | 1 | 1.32% |
|  | *Total* | *76* | *100%* |
| **Gender** | Male | 51 | 45.95% |
|  | Female | 59 | 53.15% |
|  | Prefer not to say | 1 | 0.90% |
|  | *Total* | *77* | *100%* |
| **Place** | Urban | 38 | 40.86% |
|  | Suburban | 40 | 43.01% |
|  | Rural | 15 | 16.13% |
|  | *Total* | *93* | *100.00%* |
| **Educational Attainment** | Less than high school | 1 | 1.06% |
|  | High school graduate | 1 | 1.06% |
|  | Some college | 7 | 7.45% |
|  | 2 year degree | 9 | 9.57% |
|  | 4 year degree | 14 | 14.89% |
|  | Professional degree | 35 | 37.23% |
|  | Doctorate | 18 | 19.15% |
|  | Vocational school | 1 | 1.06% |
|  | Certificate/training (please specify) | 8 | 8.51% |
|  | *Total* | *94* | *100.00%* |
